# Personalized treatment decision-making using a machine learning-derived lactylation signature for breast cancer prognosis

**DOI:** 10.3389/fimmu.2025.1540018

**Published:** 2025-05-08

**Authors:** Simin Min, Xiaonan Zhang, Yuling Liu, Weiqiang Wang, Jingwen Guan, Yuyan Chen, Meng Sun, Ziheng Wang, Tao Wang

**Affiliations:** ^1^ Clinical Research Center, Suzhou Hospital of Anhui Medical University, Suzhou, Anhui, China; ^2^ Department of Pathophysiology, Bengbu Medical University, Bengbu, Anhui, China; ^3^ Department of General Practice, Suzhou Hospital of Anhui Medical University, Suzhou, Anhui, China; ^4^ Department of Pathology, Suzhou Hospital of Anhui Medical University, Suzhou, Anhui, China; ^5^ School of Clinical Medicine, Bengbu Medical University, Bengbu, Anhui, China; ^6^ Research Laboratory Center, Guizhou Provincial People’s Hospital, Guiyang, Guizhou, China

**Keywords:** lactylation, breast cancer prognosis, machine learning, immune microenvironment, immunotherapy

## Abstract

**Background:**

Breast cancer is a heterogeneous malignancy with complex molecular characteristics, making accurate prognostication and treatment stratification particularly challenging. Emerging evidence suggests that lactylation, a novel post-translational modification, plays a crucial role in tumor progression and immune modulation.

**Methods:**

To address breast cancer heterogeneity, we developed a machine learning-derived lactylation signature (MLLS) using lactylation-related genes selected through random survival forest (RSF) and univariate Cox regression analyses. A total of 108 algorithmic combinations were applied across multiple datasets to construct and validate the model. Immune microenvironment characteristics were analyzed using multiple immune infiltration algorithms. Computational drug-repurposing analyses were conducted to identify potential therapeutic agents for high-risk patients.

**Results:**

The MLLS effectively stratified patients into low- and high-risk groups with significantly different prognoses. The model demonstrated robust predictive power across multiple cohorts. Immune infiltration analysis revealed that the low-risk group exhibited higher levels of immune checkpoints (e.g., PD-1, PD-L1) and greater infiltration of B cells, CD4^+^ T cells, and CD8^+^ T cells, suggesting better responsiveness to immunotherapy. In contrast, the high-risk group showed immune suppression features associated with poor prognosis. Methotrexate was computationally predicted as a potential therapeutic candidate for high-risk patients, although experimental validation remains necessary.

**Conclusion:**

The MLLS represents a promising prognostic biomarker and may support personalized treatment strategies in breast cancer, particularly for identifying candidates who may benefit from immunotherapy.

## Introduction

Breast cancer is the most prevalent malignancy among women and the leading cause of cancer-related mortality globally (1). The complexity and heterogeneity of breast cancer are driven not only by genetic mutations but also by metabolic alterations, which significantly impact tumor progression and treatment outcomes (2). Among these metabolic modifications, lactylation—a post-translational modification derived from lactate produced during the Warburg effect—has emerged as a critical player in breast cancer biology (3). Lactylation influences multiple aspects of cancer biology, including gene regulation, histone modification, and remodeling of the tumor microenvironment, thereby contributing to tumor growth, immune evasion, and disease progression (4). Histone lactylation particularly alters chromatin structure and transcriptional regulation, promoting oncogenic pathways and suppressing immune surveillance (5). Additionally, lactylation of non-histone targets has been implicated in modulating signaling pathways critical for cancer progression (6). Despite these advances, the precise clinical implications of lactylation in BC prognosis and personalized treatment remain incompletely understood, underscoring the need for comprehensive studies and robust predictive mode.

Recent studies have highlighted that the metabolic interplay between tumor cells and immune cells is significantly influenced by lactylation (7). Tumor cells release metabolites such as lactate, which are converted into lactylation modifications that impact nutrient availability and lead to acidosis in the tumor microenvironment (8). This acidic environment not only supports tumor cell survival but also impairs immune cell function, thereby promoting immune evasion and tumor persistence (9).

Lactylation is a key modification resulting from aberrant glycolysis in cancer cells and has received growing attention for its multifaceted roles beyond basic metabolism (10). It acts as a signaling and immunomodulatory molecule that regulates metabolic pathways, intercellular communication, and immune responses (11). During breast cancer progression, increased glycolytic activity leads to elevated lactylation levels, which accumulate in the tumor microenvironment and exacerbate its acidity (12). In addition to serving as an energy source, lactylation directly modifies histone lysine residues, thereby regulating gene expression linked to cell proliferation and tumor progression (13).

Despite the growing interest in lactylation, its specific role in breast cancer remains underexplored. This study aimed to bridge this knowledge gap by investigating the expression and functional impact of lactylation-related genes in breast cancer. We developed a machine learning-derived lactylation signature (MLLS) by integrating multiple algorithms with a ten-fold cross-validation approach, ultimately identifying seven key prognostic lactylation genes—four positively correlated with survival and two negatively correlated. This MLLS was subsequently used to evaluate immune cell infiltration, genomic instability, and potential therapeutic targets in breast cancer patients. Furthermore, we examined the relationship between MLLS and treatment response, focusing on both immunotherapy and chemotherapy, to gain deeper insights into the influence of lactylation on clinical outcomes and the tumor microenvironment.

## Methods

### Data acquisition

This study enrolled 14 breast cancer cohorts from multiple data sets, including The Cancer Genome Atlas (TCGA), Gene Expression Omnibus (GEO), Metabric, and TRANSBIG. These datasets were selected due to their comprehensive clinical annotations, extensive genomic characterization, and wide recognition in breast cancer research, enabling robust validation of our prognostic model. The specific number of cases analyzed from each dataset has been detailed as follows: TCGA-BRCA (n = 1076), GSE202203 (n = 3206), GSE96058 (n = 3409), GSE20685 (n = 327), GSE86166 (n = 330), GSE131769 (n = 298), GSE58812 (n = 107), GSE11121 (n = 200), GSE21653 (n = 244), GSE88770 (n = 108), GSE6532 (n = 87), GE20711 (n = 88), TRANSBIG (n = 198) and Metabric (n = 1747). Lactylation regulators were sourced from the published study (14).

### Lactylation signature generation and evaluation

In order to develop a lactylation-derived predictive model for breast cancer, we utilized the approach from our previous study, which incorporated ten different computational approaches (15). We generated a total of 108 combinations of these machine learning algorithms to generate a machine learning-derived lactylation signature (MLLS). Each algorithm was trained in multiple patient cohorts to get the most predictive model using the Concordance Index (C-index). Based on the RSF algorithm and univariate Cox regression analyses, seven lactylation-associated genes (Coef_ENO1_ = 0.529, Coef_RIMS1_ = 0.255, Coef_IK_ = 0.016, Coef_WBP11_ = -0.032, Coef_SF3B1_ = -0.135, Coef_CBR1_ = -0.216 and Coef_PTMA_ = -0.312) were selected. These genes served as the cornerstone for the final MLLS, which was fine-tuned to forecast patient outcomes in breast cancer.

To categorize patients, the “survminer” R package was employed. The surv_cutpoint function determined the optimal cutoff value necessary for effectively distinguishing patients into high- and low-risk classifications based on survival data. The performance of the MLLS was verified using 14 independent cohorts of breast cancer. Collectively, these cohorts represented more than 9,000 breast cancer patients, facilitating a thorough assessment of the model’s effectiveness. Furthermore, the MLLS was evaluated against 86 established breast cancer signatures, showcasing its enhanced prognostic capability across all cohorts.

### Genomic alterations in MLLS groups

Genetic variations between the high and low MLLS cohorts were investigated through the analysis of mutation levels and Copy Number Alterations (CNA), utilizing the TCGA-BRCA dataset. For patients with high and low MLLS breast cancer, Tumor Mutation Burden (TMB) was calculated from the original mutation files. The visualization of the genes with the highest mutation rates (exceeding 5%) was achieved using the maftools package. Within the TCGA-BRCA dataset, four major mutational signatures (SBS3, SBS1, SBS12, and SBS11) exhibiting increased mutation frequencies were emphasized. Moreover, the five regions most frequently subjected to amplification and deletion were identified, particularly highlighting four essential genes found in the chromosomal regions 8q24.21 and 5q21.3.

### Single-cell data processing

We applied Seurat (version 4.0) to deal with the published single-cell RNA sequence (scRNA-seq) from the GEO database (GSE161529) (16). This procedure included the elimination of genes with no detected expression while preserving those displaying non-zero expression levels. Seurat’s “SCTransform” function was utilized to normalize the expression matrix. Dimensionality reduction was achieved via principal component analysis (PCA) and Uniform Manifold Approximation and Projection (UMAP). To identify cell clusters, we applied Seurat’s “FindNeighbors” and “FindClusters” functions. In order to maintain the dataset’s integrity and reliability, potential doublets were eliminated using the DoubletFinder package (17). Cells not passed quality control—such as having mitochondrial gene content exceeding 15% or showing fewer than 500 expressed genes—were excluded from the analysis. Finally, 25,605 cells remained for further analysis. The final identification of 20 clusters was justified by evaluating the stability and reproducibility across multiple resolutions (0.2 to 1.0), selecting the resolution that best balanced detailed cellular heterogeneity and interpretability in the context of breast cancer biology. Cell types were identified through manual annotation, relying on recognized marker genes.

### Inference of gene regulatory networks and regulon clustering

SCENIC methodology was utilized to build gene regulatory networks (GRNs) using scRNA-seq data as we reported before (15). Briefly, we utilized transcription factors (TFs)-target pairs to identify co-expression modules and confirmed the direct target genes in each module. The regulatory activity score (RAS) for every cell is computed by evaluating the area beneath the recovery curve. Moreover, data were transformed into the metacells to improve data quality and minimized computational requirements (18).

We clarified the regulatory dynamics between TF-target pairs, with a specific emphasis on the clustering of TFs. First, the data regarding TF-target interactions were refined to retain only those pairs that exceeded a specified significance threshold (>1), ensuring that the most pertinent regulatory interactions were highlighted (19). The following analyses focused on pinpointing chief TFs by evaluating the depth of their influence on target genes, marking them as crucial nodes within the GRNs.

### Analysis of TME differences and immunotherapy outcomes

To thoroughly evaluate levels of immune cell infiltration, we calculated six immune infiltration algorithms using IOBR package to analyze the presence of adverse tumor microenvironment (TME) classified by the MLLS (20). We further assessed the ESTIMATE and TIDE, which offered essential insights regarding the potential for immunotherapy for breast cancer patients (21, 22). Additionally, immune checkpoints were measured as indicators of the immune condition and acted as initial predictors of how patients might respond to immune checkpoint inhibitors (ICIs) therapy.

### Identification of therapeutic drugs for high-risk MLLS patients

To explore the potential therapeutic targets for high MLLS breast cancer patients, we got 6,125 micromolecules from the Drug Repurposing Hub. Spearman correlation was calculated to the MLLS riskscore with gene expression (coefficients > 0.3 and P-value < 0.05) and CRES scores (coefficients < -0.3 and P-value < 0.05). Furthermore, CTRP and PRISM databases were utilized to assess the drug responsiveness respectively. Finally, the Connectivity Map (CMap) database was executed to identify the most promising therapeutic agents (15). CMap score < -95 was indicative of a greater therapeutic potential against breast cancer in this study.

### Sample collection and immunohistochemistry

A total of 30 breast cancer patient samples were collected from Guizhou Provincial People’s Hospital. Tumor tissues were confirmed by hematoxylin and eosin (HE) staining to ensure the presence of cancerous cells. The inclusion criteria for patient selection were based on clinical diagnosis, and informed consent was obtained from all participants prior to sample collection.

The expression levels of seven key genes previously identified in our MLLS model were measured using qPCR. The MLLS model classifies patients based on the expression profiles of these genes, with a focus on identifying signatures associated with prognosis and treatment response in breast cancer. The expression data were used to categorize the patients into distinct risk groups as per the model. Immunohistochemical staining was conducted on formalin-fixed, paraffin-embedded tumor tissue sections. The staining procedure and antibody selection followed protocols described in our previously published work (23, 24).

## Results

### Development of a machine learning-derived lactylation signature for breast cancer prognosis

**Figure 1:**
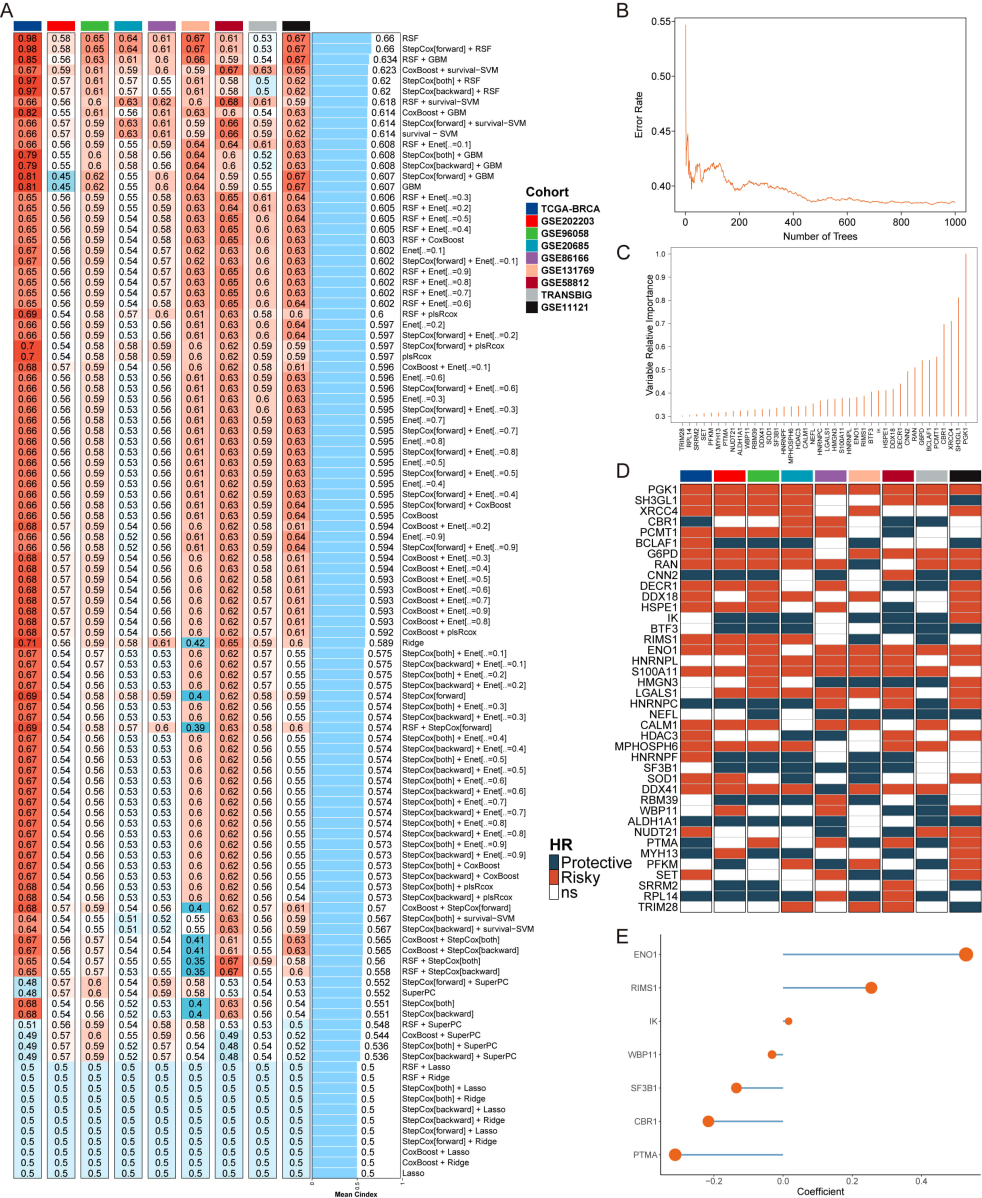
Development of a machine learning-derived lactylation signature for breast cancer prognosis. **(A)** Average C-index of 108 combination algorithms in 9 breast cancer cohorts. **(B)** Error rate of the RSF in 1000 iterations. **(C)** Importance of top genes. **(D)** Prognosis of top genes in 9 breast cancer cohorts **(E)**. Correlation coefficients of key genes used in model.

In this research, we utilized lactylation-associated genes to develop a machine learning-based lactylation signature (MLLS) aimed at establishing a prognostic model for breast cancer patients. By integrating 108 distinct combinations of machine learning methods within a ten-fold cross-validation framework, we sought to identify the most effective predictive model for patient survival. We calculated the average C-index for each algorithm combination within the TCGA-BRCA cohort, as well as across eight independent validation cohorts. The Random Survival Forest (RSF) approach, which achieved the highest average C-index of 0.66, was selected to evaluate the predictive efficacy of the model (**Figure 1A**). To identify key lactylation-related genes, we conducted 1000 random forest tests, revealing genes associated with the minimal error rate (**Figure 1B**). We then constructed a relative variable importance plot to illustrate the contributions of these genes to the model (**Figure 1C**). Additionally, we employed univariate Cox regression analysis to evaluate the prognostic significance of the selected lactylation-related genes, calculating hazard ratios (HRs) across the nine cohorts (**Figure 1D**).

From this analysis, two positively correlated genes (ENO1 and RIMS1) and five negatively correlated genes (IK, WBP11, SF3B1, CBR1 and PTMA) were identified and used to construct the MLLS model (**Figure 1E**). These genes are involved in critical biological pathways such as angiogenesis, nutrient transport, and circadian rhythm regulation, which are implicated in cancer progression and treatment response. By incorporating these genes, we aimed to enhance the model’s prognostic utility, particularly in differentiating patient outcomes based on lactylation profiles.

To assess the efficacy of the MLLS model, we determined risk scores for each sample within the nine cohorts. The MLLS successfully categorized patients into high-risk and low-risk groups (**Supplementary Figure S1A**). The Kaplan-Meier survival analysis indicated that patients identified as high-risk demonstrated a notably lower survival rate than those in the low-risk group (**Supplementary Figure S1B**).

Given the known molecular heterogeneity of breast cancer, we evaluated the performance of our MLLS model across distinct molecular subtypes. Our analyses did not identify significant subtype-specific differences in MLLS prognostic performance, indicating the robustness and broad applicability of the lactylation signature across diverse breast cancer contexts.

### 87 Comprehensive evaluation of MLLS predictive model with published breast cancer signatures

To further assess the predictive accuracy and reliability of the MLLS model, we conducted a comparison with 87 previously established prognostic models in breast cancer across nine distinct cohorts. Both univariate and multivariate Cox analyses revealed that the MLLS acted as an independent risk factor when evaluated against other clinical indicators, such as age, menopause status, and disease stage (**Supplementary Figure S2A**). By employing three variables—MLLS risk score, age, and stage (with stage included due to its prevalent clinical usage despite the absence of statistical significance)—we created a nomogram to estimate patients’ survival probabilities at 1, 3, and 5 years (**Supplementary Figure S2B**). The nomogram’s forecasts for overall survival (OS) among patients with different breast cancer types were consistent with the actual survival rates observed in the entire cohort, as demonstrated by calibration curves and decision curve analysis (DCA) (**Supplementary Figures S2C-E**). This alignment emphasizes the enhanced capability of the nomogram in predicting patient outcomes. Additionally, the area under the receiver operating characteristic curve (AUC) for the MLLS model (0.66) exceeded that of other clinical variables, signifying that the MLLS risk model was superior in predicting patient outcomes (**Supplementary Figure S2F**).

**Figure 2:**
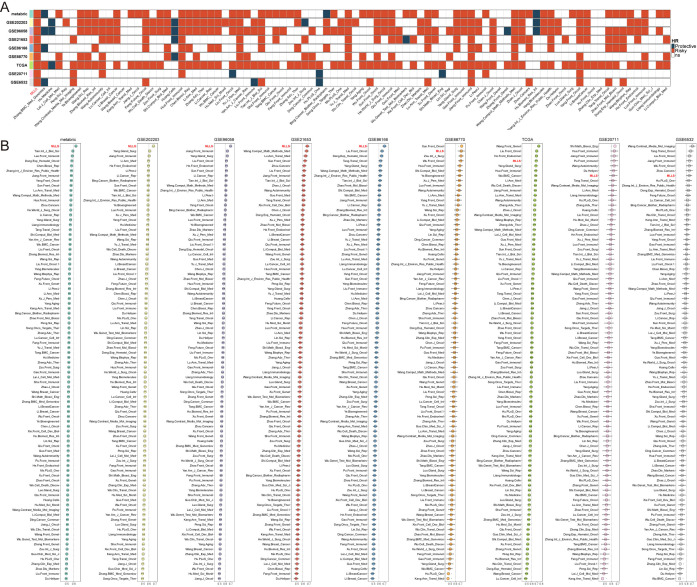
Comprehensive evaluation of MLLS predictive model with 87 published breast cancer signatures. **(A)** univariate Cox analysis of models in 10 BC cohorts. **(B)** Comparison of the average C-index of models in 10 breast cancer cohorts.

To enhance the assessment of the predictive capabilities and consistency of the MLLS model, we gathered and analyzed 86 models that had been previously published, spanning nine distinct cohorts. Among these models, the MLLS was the only one that exhibited statistical significance across all nine cohorts (**Figure 2A**). We evaluated the average C-index for each model by using varied datasets to measure stability. The findings revealed that the MLLS model reliably ranked among the top models in every cohort, securing first place in five of them, second in one, fourth in another, and seventh in two. This performance highlights the impressive robustness and superior effectiveness of the MLLS model when compared to its peers (**Figure 2B**).

### Genetic alteration landscape associated with MLLS

**Figure 3:**
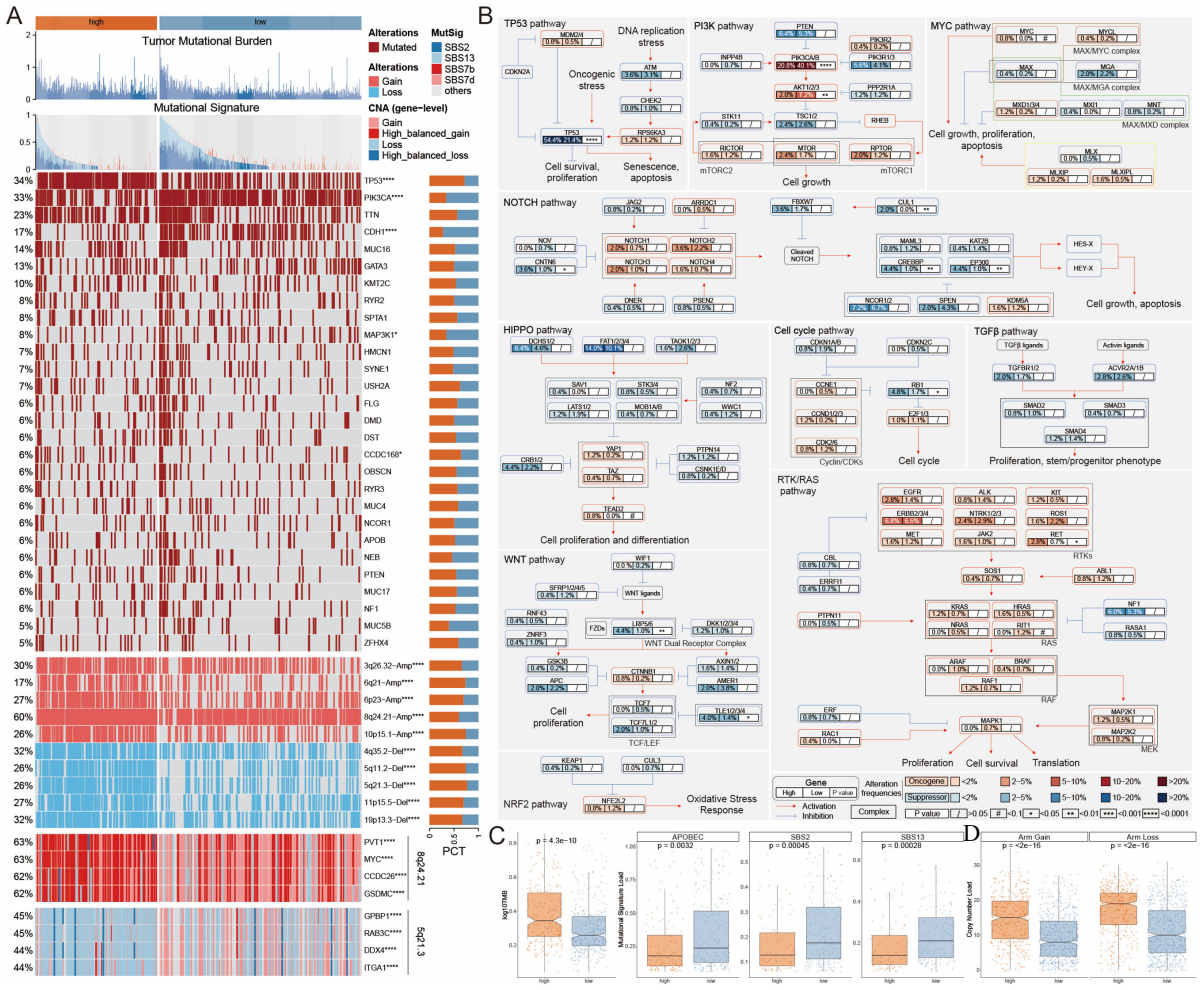
Genetic alteration landscape associated with MLLS. **(A)** Genomic alteration landscape of MLEM, from up to bottom: TMB, gene mutational signatures, gene mutation frequency, CNAs (the red represents amplification, and the blue represents deletion), and the representative genes in region 8q24.21 and 5q21.3. **(B)** Mutation frequency of 10 oncogenic pathways between MLLS groups. **(C)** Comparison of TMB between MLEM groups. **(D)** Amplification or deletion of chromosomal arm. *P<0.05, **P<0.01, ***<0.001, ****P<0.0001.

The genetic landscape of tumor cells shows notable variability among different patients. To explore the genetic diversity between high and low MLLS cohorts, we examined gene mutations as well as copy number alterations (CNAs) in each group. Our initial assessment of TMB indicated that individuals in the high MLLS cohort had a TMB that surpassed that of their low MLLS counterparts (**Figures 3A, C**). Furthermore, we explored CNAs, where red denotes copy number gains and blue denotes losses. The findings demonstrated that the high MLLS cohort exhibited more significant amplifications and deletions at the chromosome arm level (**Figures 3A, C**). This included the amplification of specific regions like 3q26.32, 6q21, 6p23, 8q24.21, and 10p15.1, alongside deletions in regions such as 4q35.2, 5q11.2, 5q21.3, 11p15.5, and 19p13.3 (**Figure 3A**). Noteworthy is that genes including PVT1, MYC, CCDC26, and GSDMC located on chromosome 8q24.21 were amplified significantly, while GPBP1, RAB3C, DDX4, and ITGA1 on chromosome 5q21.3 demonstrated significant deletions (**Figure 3A**).

By synthesizing information from the TCGA database related to ten established cancer signaling pathways, we discovered that classical tumor suppressor genes such as TP53, CNTN6, CREBBP, and EP300RB1 had a higher frequency of mutations in the high MLLS group. In contrast, oncogenes like PIK3CA/B, AKT, and RET exhibited more prevalent mutations in the low MLLS group (**Figure 3B**). Additionally, mutation signatures such as SBS2, SBS13, and APOBEC were markedly lower in the high MLLS group (**Figure 3C**). In conclusion, the increased TMB along with heightened deletions and amplifications at the chromosome arm level in the high MLLS group may play a significant role in contributing to poor prognostic outcomes (**Figure 3D**).

### Single-cell analysis reveals transcriptional and regulatory mechanisms associated MLLS

**Figure 4:**
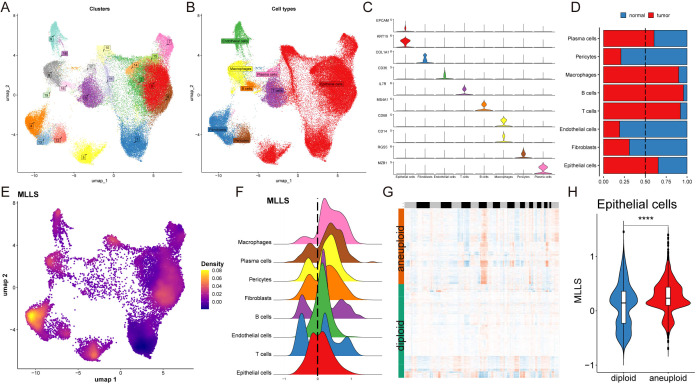
Single-cell analysis reveals biological mechanisms underlying MLLS. **(A)** UMAP visualization illustrates the distribution of cell clusters. **(B)** UMAP visualization illustrates the distribution of identified cell types. **(C)** Representative markers of each cell type. **(D)** Proportion of eight cell types between tumor and normal tissues. **(E)** UMAP visualization illustrates the distribution of MLLS value. **(F)** Distribution of MLLS value across various cell types. **(G)** Estimation of copy number using copyKAT algorithm. **(H)** MLLS variance between diploid and aneuploid cells in the epithelial cell. ****P<0.0001.

The properties of the MLLS were further examined at the single-cell level. We chose 15 patients, consisting of 6 with normal tissue and 9 with breast cancer tumor tissue, for an in-depth assessment of MLLS (**Supplementary Figures S3A, B**). The cells were divided into 20 clusters and 8 unique cell types (**Figures 4A, B**). We measured the quantity of cells for each type and evaluated the proportion of each cell type across the patients (**Supplementary Figures S3C, D**). Cells were marked with specific representative markers corresponding to each cell type, and the actual distribution of these markers was scrutinized (**Figure 4C**; **Supplementary Figure S3E**). Single-cell sequencing identified transcriptomic variations among cell types between normal and tumor tissue. The findings indicated significant infiltration of macrophages, plasma cells, B cells, T cells, and epithelial cells within the tumor tissue (**Figure 4D**). The MLLS model was employed for single-cell analysis to create a detailed cell distribution map (**Figure 4E**), with epithelial cells further grouped into high and low MLLS categories based on peak scores (**Figure 4F**).

After this classification, we performed differential gene expression analysis and functional clustering for the 8 identified cell types to elucidate potential functional pathways (**Supplementary Figures S3F, G**). To assess copy number alterations and to distinguish tumor cells from normal epithelial cells, the CopyKAT package was utilized (**Figure 4G**). Our study demonstrated that tumor-aneuploid cells had a greater MLLS score than tumor-diploid cells, highlighting the pivotal role of MLLS in breast cancer progression (**Figure 4H**).

### Identification of regulatory factors influencing MLLS and cellular differentiation

**Figure 5:**
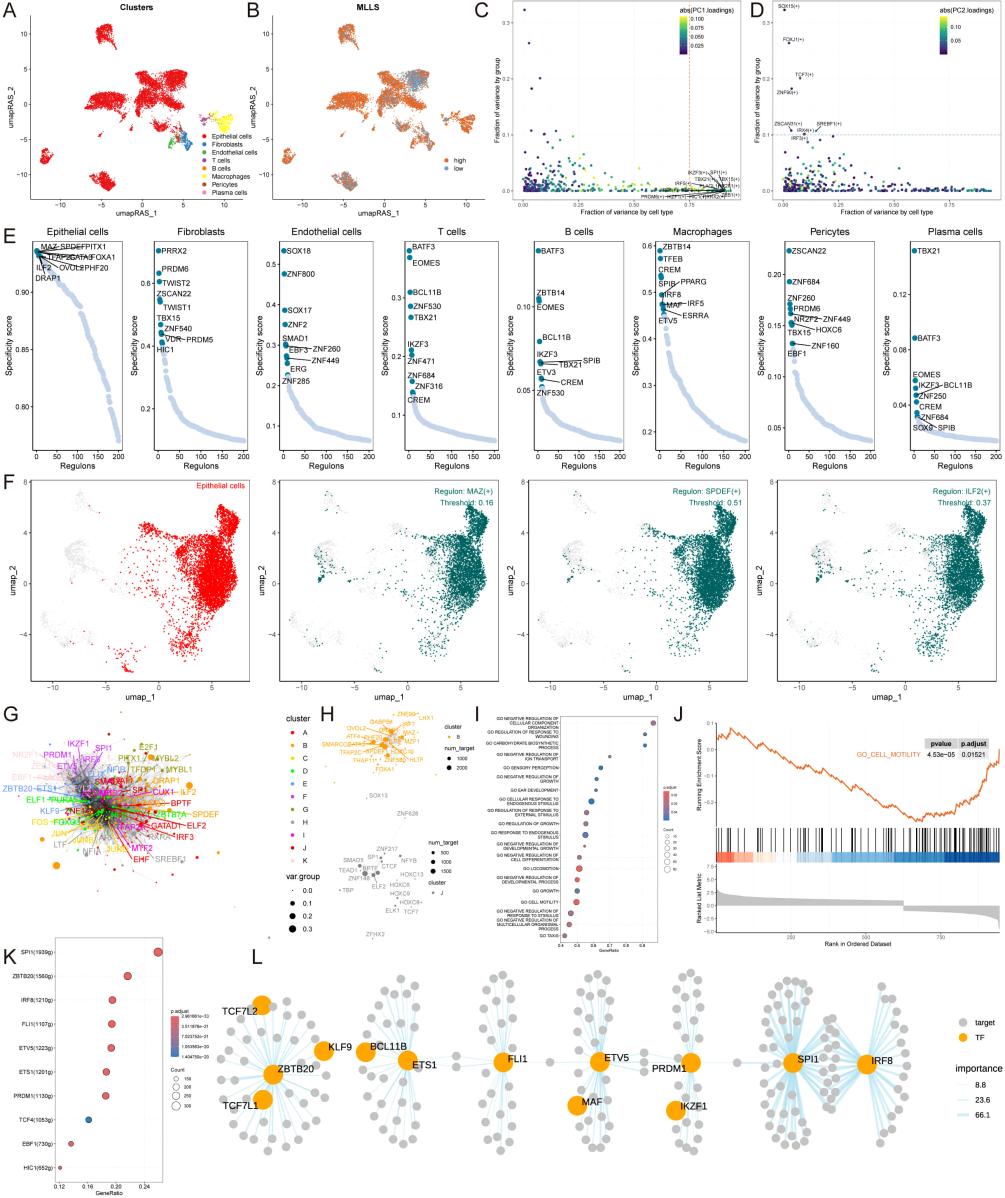
Identification of regulatory factors influencing MLLS and cellular differentiation. **(A)** umapRAS visualization illustrates the distribution of cell clusters. **(B)** umapRAS visualization illustrates the distribution of MLLS. **(C)** Variance analysis plot highlights the PC1 impact of cell types. **(D)** Variance analysis plot highlights the PC2 impact of MLLS. **(E)** Regulons ranking for each cell type based on RSS. **(F)** Three top regulons focus on epithelial cells. **(G)** Interactions network of regulons constructed using the Leiden algorithm. **(H)** Detail network of modules B and **(J, I)** Functional variations linked to MLLS in epithelial cells. **(J)** Representative pathways in the context of high MLLS. **(K)** TFs involved in cell motility. **(L)** Detailed regulatory network of the interactions among TFs involved in cell motility.

To gain a deeper understanding of the regulatory mechanisms underlying MLLS, we utilized the SCENIC pipeline to construct gene regulatory networks from single-cell RNA sequencing data, incorporating cis-regulatory sequence information. The gene expression data were transformed into RAS for TFs (**Figures 5A, B**). Principal component analysis (PCA) and variance decomposition were subsequently performed. PCA1 revealed TFs specific to cell types, while PCA2 highlighted TFs specific to MLLS (**Figures 5C, D**).

Using Jensen-Shannon divergence, we identified the top 10 key TFs for each cell type based on the specific scores of each regulator. For epithelial cells, we focused on the top three regulatory factors with the highest regulon specificity scores (RSS)—MAZ, SPDEF, and ILF2—as the most relevant regulators, and we conducted similar analyses for the other seven cell types (**Figures 5E, F**; **Supplementary Figure S4A**).

To elucidate the cooperative relationships among TFs in regulating specific biological functions in MLLS, we analyzed RAS scores for each regulatory pair using the Leiden algorithm. This cluster analysis identified eleven TF clusters, with Clusters B and J having the highest contributions to MLLS development (**Figures 5G, H**; **Supplementary Figure S4B**). Gene Set Enrichment Analysis (GSEA) of epithelial cells revealed the activation of several pathways, while the cell motility pathway was notably inhibited in cells with low MLLS (**Figures 5I, J**). Further identification of TFs involved in regulating cell motility and influencing MLLS progression was performed, resulting in a regulatory network diagram depicting the relationships among these TFs (**Figures 5K, L**).

### Immune profiling and identification of immunotherapeutic targets in MLLS

**Figure 6:**
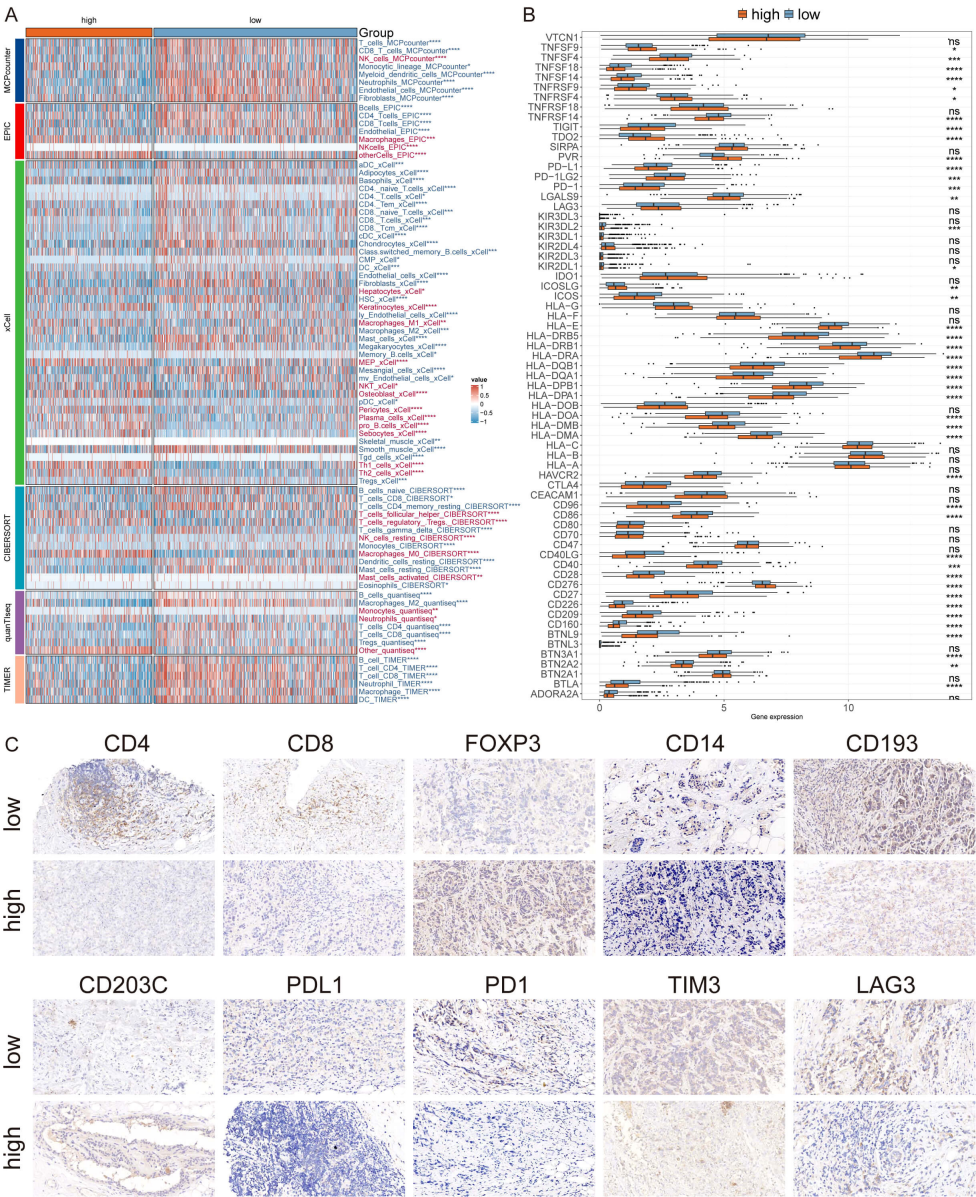
Differential expression and immunohistochemical analysis of immune markers in tumor microenvironments between MLLS subgroups. **(A)** Heatmap providing a comparative view of immune cell infiltration in tumor samples with low and high MLLS, utilizing various computational algorithms for quantification. Each row represents a different type of immune cell, with the color intensity reflecting the level of infiltration. Red text indicates increased infiltration in the high MLLS group, while blue text indicates decreased infiltration. **(B)** Box plots illustrating the distribution of gene expression levels for ICIs across low versus high MLLS conditions, with statistical significance denoted by ns for not significant; *P < 0.05; **P < 0.01; ***P < 0.001; ****P < 0.0001. **(C)** Representative immunohistochemistry images showcasing the staining intensity of various immune markers between high and low expression conditions, visually depicting the differential expression of these markers in correlation with MLLS levels.

To assess potential immunotherapeutic targets in patients categorized by high and low MLLS, we utilized six different algorithms to evaluate immune cell infiltration within breast cancer patients. The findings indicated that individuals in the low MLLS category displayed considerably greater levels of immune cell infiltration, comprising CD4^+^ T cells, CD8^+^ T cells, B cells, NK cells, and monocytes, when compared to those in the high MLLS category (**Figure 6A**). Additionally, expression levels of critical ICIs, including PD-L1, PD-1, CTLA4, and HAVCR2, were markedly higher in the low MLLS group, implying an enhanced sensitivity to immunotherapy in these individuals (**Figure 6B**). Immunohistochemistry (IHC) supported these conclusions through the use of representative cell markers and clinical ICIs (**Figure 6C**).

**Figure 7:**
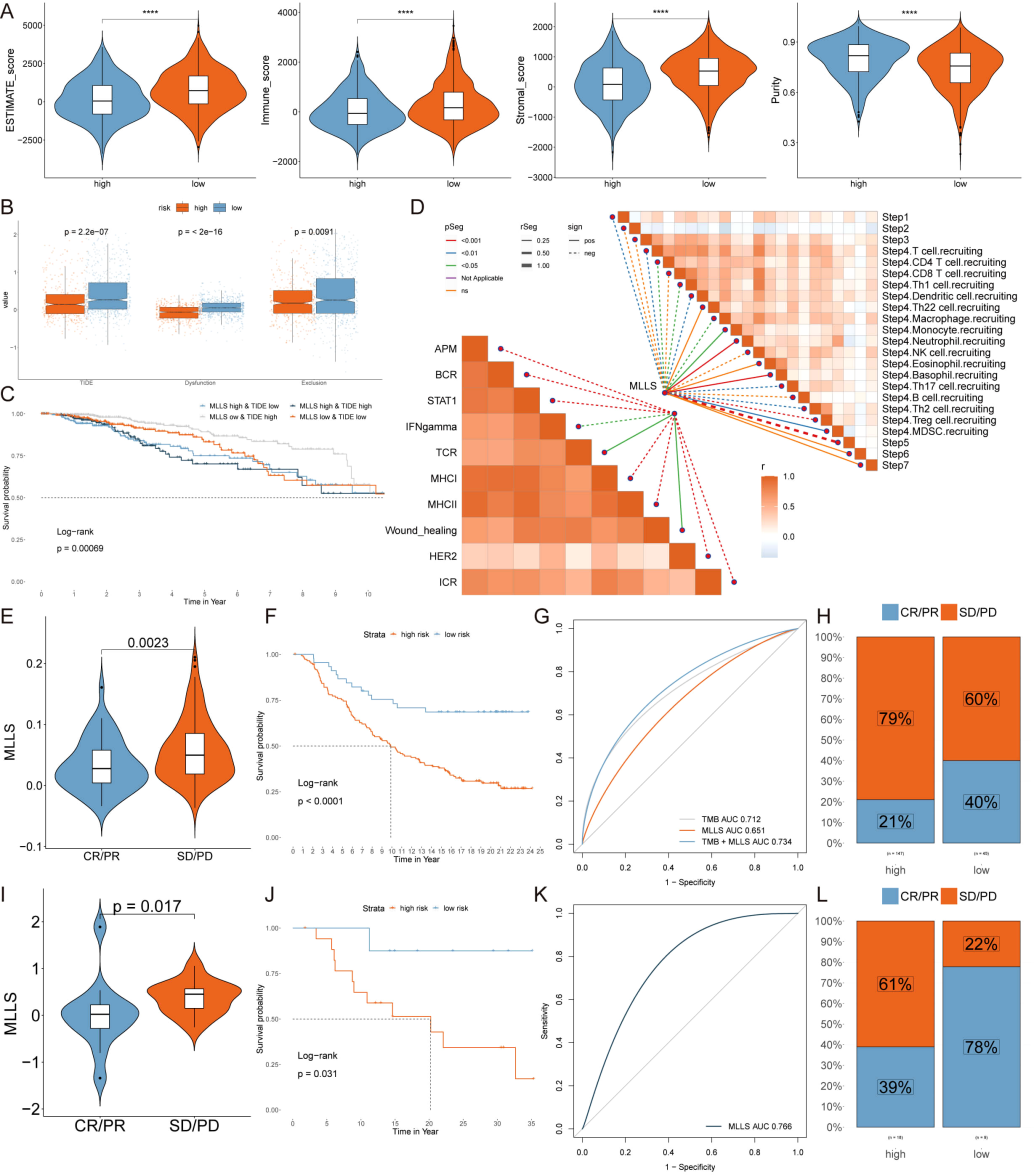
Immune profiling and identification of immunotherapeutic targets in MLLS. **(A)** ESTIMATE scores, immune scores, stromal scores, and tumor purity between MLLS groups. **(B)** TIDE, dysfunction, and exclusion variations between MLLS groups. **(C)** Survival probability of patients based on the combination of MLLS and TIDE. **(D)** Correlation analysis of MLLS with immune pathways and tumor immune cycle. **(E, I)** Violin charts display the relationship between MLLS levels and responses to anti-PDL1 **(E)** and anti-PD1 **(I)** therapies, detailing the differential immune responses. **(F, J)** Survival probabilities of low and high MLLS patients in anti-PDL1 **(F)** and anti-PD1 **(J)** cohorts, respectively, illustrating the impact of MLLS on survival outcomes. **(G, K)** Analysis estimates the predictive ability of MLLS via AUC values, considering TMB combinations, in anti-PDL1 **(G)** and anti-PD1 **(K)** cohorts, evaluating the efficacy of MLLS as a biomarker. **(H, L)** The percentages of complete response/partial response (CR/PR) and stable disease/progressive disease (SD/PD) in anti-PDL1 **(H)** and anti-PD1 **(L)** cohorts are shown, based on MLLS levels, to assess treatment effectiveness.

Subsequently, we applied the ESTIMATE algorithm to assess tumor immune microenvironments, revealing that the ESTIMATE, immune, and stromal scores were elevated, whereas tumor purity was diminished in the low MLLS cohort (**Figure 7A**). Moreover, the low TIDE, Exclusion, and Dysfunction scores noted in the high MLLS group suggested an increased likelihood of immune evasion, potentially influencing the diminished effectiveness of ICI therapy (**Figure 7B**). The Kaplan-Meier analysis showed that patients with low MLLS and elevated TIDE scores had prolonged survival compared to other group combinations (**Figure 7C**). Taken together, these findings suggest that individuals with low MLLS exhibit enhanced anti-tumor immune activity relative to those with high MLLS (**Figure 7D**).

To further investigate the ability of MLLS to predict responses to immune checkpoint blockade therapy, we analyzed data from both the anti-PD-L1 cohort (IMvigor210) and the anti-PD-1 cohort (GSE78220). Patients with low MLLS demonstrated notable therapeutic benefits and clinical improvements in both cohorts (IMvigor210: **Figures 7E–H**; GSE78220: **Figures 7I–L**).

### Identification of potential therapeutic agents for high MLLS patients

**Figure 8:**
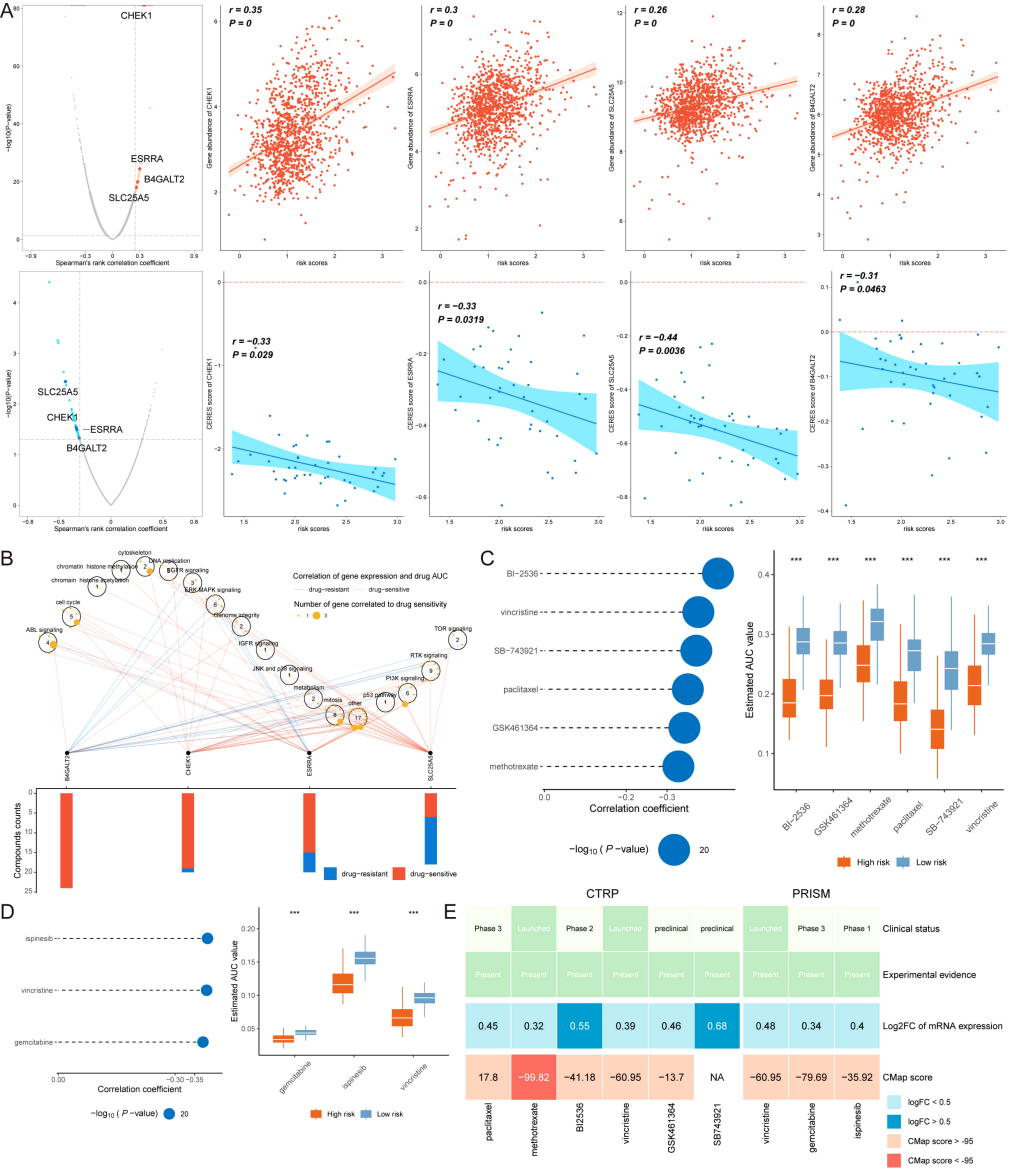
Identification of potential therapeutic agents for high MLLS patients. **(A)** Spearman’s correlation illustrating the association between MLLS and the abundance of potential therapeutic targets in breast cancer patients. **(B)** Network analysis highlights the intricate connections between these therapeutic targets and their associated drug action pathways. **(C)** Box plots compare the AUC values of 6 compounds in the CTRP dataset. **(D)** Box plots compare the AUC values of 3 compounds in the PRISM dataset. **(E)** Summary table outlines the multi-perspective analysis of the 9 candidate compounds, detailing their clinical status, experimental evidence, mRNA expression levels, and CMap scores.

Cancer treatment often involves chemotherapy as a standard approach. In our research, we leveraged data from various datasets to identify potential targeted therapies for patients with breast cancer who present elevated MLLS scores. Our findings indicated a positive relationship between the MLLS scores and the expression levels of four promising therapeutic targets: CHEK1, ESRRA, B4GALT2, and SLC25A5. In contrast, we noted a negative relationship with their CERES scores, indicating a potential vulnerability among patients exhibiting high MLLS scores (**Figure 8A**). Additionally, these targets were associated with several essential drug action pathways, highlighting their importance as vital therapeutic targets for this particular group of patients (**Figure 8B**).

From the CTRP dataset, we identified six compounds (BI-2536, GSK461364, methotrexate, paclitaxel, SB-743921, and vincristine), and from the PRISM dataset, we identified three compounds (gemcitabine, ispinesib, and vincristine). Patients in the high MLLS group showed lower AUC values for these compounds compared to the low MLLS group, suggesting increased sensitivity to these chemotherapeutic agents (**Figures 8C, D**). The clinical status, experimental evidence, mRNA expression levels, and CMap scores for each compound were further assessed through CMap analysis, leading to the identification of methotrexate as the most favorable treatment for patients with high MLLS, given its CMap score of -99.82 (**Figure 8E**).

## Discussion

Breast cancer is a highly heterogeneous malignancy originating in breast tissue (25). Despite advancements in early detection and therapeutic approaches, clinical outcomes for breast cancer patients remain suboptimal. As such, there is an urgent need to identify novel molecular markers that can improve prognostication and patient management. Machine learning has emerged as a promising tool for efficiently predicting relapse risk using genetic signatures, offering new avenues for personalized medicine. Previous studies have demonstrated that genetic characteristics can provide essential prognostic information and improve risk assessment, and these approaches have already been integrated into clinical guidelines (26, 27).

In this study, we focused on lactylation, a post-translational modification that occurs due to lactate accumulation, which has been shown to play a crucial role in tumorigenesis. Lactylation modulates protein functions, such as histone lysine lactylation (Kla), which directly affects gene expression and cellular processes like immune evasion, cell communication, and drug sensitivity, all of which influence tumor progression (5, 28, 29). For instance, lactylation of MOESIN has been shown to modulate interactions with TGF-β receptors, affecting cancer development (6). The present study aimed to explore these dynamics and provide new insights into breast cancer progression by developing a lactylation-based prognostic model.

The selected lactylation-related genes for model construction have been implicated in distinct biological processes relevant to breast cancer progression. For example, ENO1, a critical glycolytic enzyme, may influence lactate production and thus lactylation modification (30, 31). WBP11 could affect tumor cell proliferation and metabolic reprogramming (32). However, direct evidence linking RIMS1, IK, SF3B1, and CBR1 genes specifically to lactylation modifications in breast cancer is lacking. Given that these genes are known to be involved in transcriptional regulation, RNA splicing, or immune functions, they may theoretically influence tumor progression indirectly through the modulation of lactylation-related pathways. Further experimental validation is required to clearly elucidate these potential indirect mechanisms.

The MLLS model provided a novel approach to predicting breast cancer prognosis by integrating lactylation-related genes and machine learning algorithms. We employed the RSF algorithm, which exhibited the highest C-index, to construct the model. This approach demonstrated robust predictive performance in both training and test datasets. Furthermore, we applied six algorithms to evaluate immune cell infiltration in breast cancer patients, revealing distinct immune responses and clinical outcomes between high- and low-risk groups. These findings suggest that the MLLS model is a reliable predictor of immune infiltration and therapeutic response, indicating its potential for improving personalized treatment strategies.

While the findings largely align with the current understanding of lactylation’s role in cancer, there were a few unexpected discoveries that shed new light on the role of lactylation in breast cancer. One of the most surprising results was the inverse correlation between MLLS scores and immune infiltration in breast cancer patients. High MLLS patients exhibited lower immune cell infiltration and increased immune suppression, which is contrary to the common association between higher lactate levels and immune activation. Lactylation, particularly histone lactylation, has previously been linked to immune evasion (5). Our study suggests that lactylation could be acting as a mechanism for immune suppression, reducing immune cell infiltration and contributing to tumor immune evasion. This finding challenges the conventional view of lactylation as a purely metabolic modifier and introduces the concept of lactylation as a modulator of the immune microenvironment. This immune-suppressive role of lactylation could have important implications for immunotherapy in breast cancer, suggesting that targeting lactylation may enhance immune responses against the tumor. Lactylation, initially recognized primarily as a histone modification, contributes to immune suppression by reshaping the transcriptional landscape of immune-related genes. Histone lactylation, particularly at lysine residues, has been shown to alter chromatin accessibility, leading to transcriptional activation of genes involved in tumor immune evasion (5, 28). Recent findings further reveal that lactylation also targets non-histone proteins, such as MOESIN, modulating TGF-β signaling pathways and directly influencing immune regulatory cells like Tregs (6). Therefore, lactylation likely suppresses immune infiltration through dual mechanisms—epigenetic regulation via histone modifications and direct functional modulation via non-histone protein lactylation. Further experimental studies are essential to fully dissect these intricate mechanisms.

Another unexpected discovery was the association between high MLLS and chemoresistance. Although lactate is often implicated in drug resistance due to its effects on cellular metabolism and acidification of the tumor microenvironment, the specific contribution of lactylation to this process was not well understood. Our study suggests that lactylation-related genes could play a direct role in chemoresistance, particularly in chemotherapy-resistant breast cancer. This aligns with recent findings indicating that metabolic reprogramming, including lactate accumulation, contributes to treatment failure in cancer (33). However, the mechanism by which lactylation confers resistance requires further investigation, as it might be linked to alterations in drug uptake or activation of survival pathways in tumor cells.

A surprising finding was the lack of correlation between MLLS and TMB in some subgroups. While TMB is typically associated with poor prognosis and immune response in many cancers, including breast cancer, our study revealed that low MLLS patients did not necessarily have higher TMB, yet they still showed better immune infiltration and therapeutic response. This discrepancy suggests that lactylation may function independently of genetic mutations and that non-genomic factors such as epigenetic modifications and immune modulation could play a more dominant role in lactylation-related tumor progression. Rather than relying solely on pathway enrichment analysis, which yielded inconsistent results due to the diverse mutation profiles, we integrated previously reported functional roles of commonly mutated genes. For example, TP53 mutations are widely documented to enhance glycolytic activity, subsequently increasing lactate availability for lactylation modifications, thus providing a plausible mechanistic link to our observed lactylation signature. On the other hand, the metabolic consequences of PIK3CA mutations differ significantly, potentially explaining their association with distinct lactylation statuses.

Another surprising aspect was the role of lactylation in regulating the TME. While lactate accumulation is known to influence TME properties, our study found that lactylation itself may directly modulate immune cell composition and immune checkpoint expression. High MLLS patients exhibited increased proportions of immune-suppressive cell types, such as M0 macrophages and neutrophils, while low MLLS patients showed higher proportions of T cells. This suggests that lactylation could be a key factor in immune evasion within the TME and might provide a new target for therapies aimed at modulating the immune microenvironment.

Our study provides novel insights into the role of lactylation in breast cancer progression and therapeutic response. These findings suggest that lactylation-related genes not only serve as prognostic biomarkers but also play a crucial role in immune modulation and chemoresistance. The MLLS model could become a valuable tool for identifying patients who are at high risk of immune suppression and chemoresistance, thereby informing treatment strategies such as immunotherapy and targeted therapies. Our study’s novelty lies specifically in the focus on lactylation—a unique post-translational modification driven by lactate accumulation distinct from general metabolic signatures or hypoxia-induced models. Unlike broad metabolic or hypoxia models primarily reflecting general tumor metabolic states or oxygen deprivation, our lactylation-based signature directly captures specific epigenetic and non-histone protein modifications impacting immune evasion, tumor progression, and treatment resistance. By targeting these lactylation-specific processes, our model provides unique prognostic value and potentially actionable targets beyond those identified by generalized metabolic or hypoxia signatures.

Although our findings computationally link lactylation to immune suppression and chemoresistance, direct experimental validation was beyond the scope of this study. Future *in vitro* and *in vivo* experiments, including CRISPR-based gene editing and overexpression systems, are necessary to establish causality and to elucidate precisely how lactylation-related genes influence immune checkpoint expression and drug resistance mechanisms. Furthermore, methotrexate, identified via computational analysis as potentially effective for patients with elevated lactylation signatures, is traditionally recognized as an inhibitor of dihydrofolate reductase (DHFR). While methotrexate does not directly target lactylation enzymes, it likely exerts indirect effects by altering metabolic pathways essential for nucleotide synthesis, potentially disrupting lactate production and lactylation-related metabolic processes. Additional preclinical and clinical studies are required to experimentally confirm the therapeutic relevance of methotrexate in patients stratified by lactylation profiles.

Our study demonstrates robust predictive performance using large-scale retrospective datasets, several limitations should be acknowledged. First, the analyses rely solely on retrospective data, inherently carrying risks of selection and reporting biases. Second, while extensive computational validation was conducted, prospective clinical validation in independent cohorts remains essential to confirm the clinical utility and generalizability of our findings. Future prospective and experimental studies are necessary to solidify the clinical application of the MLLS model.

## Conclusion

This study highlights the potential of lactylation-based biomarkers in predicting prognosis and treatment response in breast cancer. The unexpected findings regarding lactylation’s role in immune suppression and chemoresistance suggest that targeting lactylation could provide new therapeutic opportunities, particularly in patients who are resistant to conventional therapies. Future research will need to address the functional mechanisms of lactylation in the tumor microenvironment and immune modulation to fully realize its potential as a therapeutic target in breast cancer.

## Data Availability

All data used in this study were sourced from the public databases online or may be made available from the corresponding author upon reasonable request.
