# Supplementary material for: Personalized treatment decision-making using a machine learning-derived lactylation signature for breast cancer prognosis
Source: Front Immunol. 2025 May 8;16:1540018. doi: 10.3389/fimmu.2025.1540018 (PMC12095166; doi:10.3389/fimmu.2025.1540018)
Supplement: Supplementary file 1 [file DataSheet1.pdf]

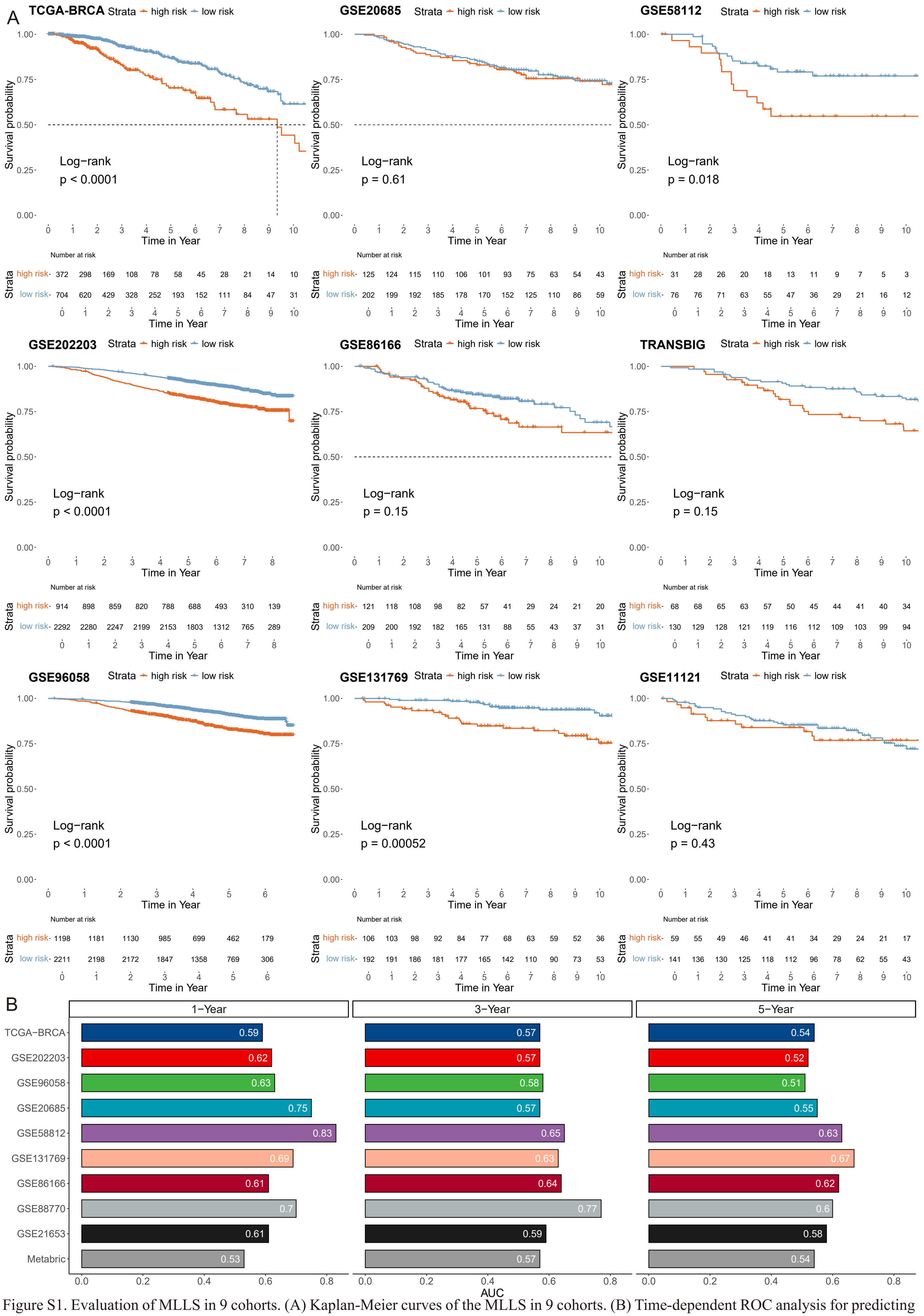

Figure S1. Evaluation of MLLS in 9 cohorts. (A) Kaplan-Meier curves of the MLLS in 9 cohorts. (B) Time-dependent ROC analysis for predicting OS at 1, 3, and 5 years.
