# Supplementary material for: Personalized treatment decision-making using a machine learning-derived lactylation signature for breast cancer prognosis
Source: Front Immunol. 2025 May 8;16:1540018. doi: 10.3389/fimmu.2025.1540018 (PMC12095166; doi:10.3389/fimmu.2025.1540018)
Supplement: Supplementary file 2 [file DataSheet2.pdf]

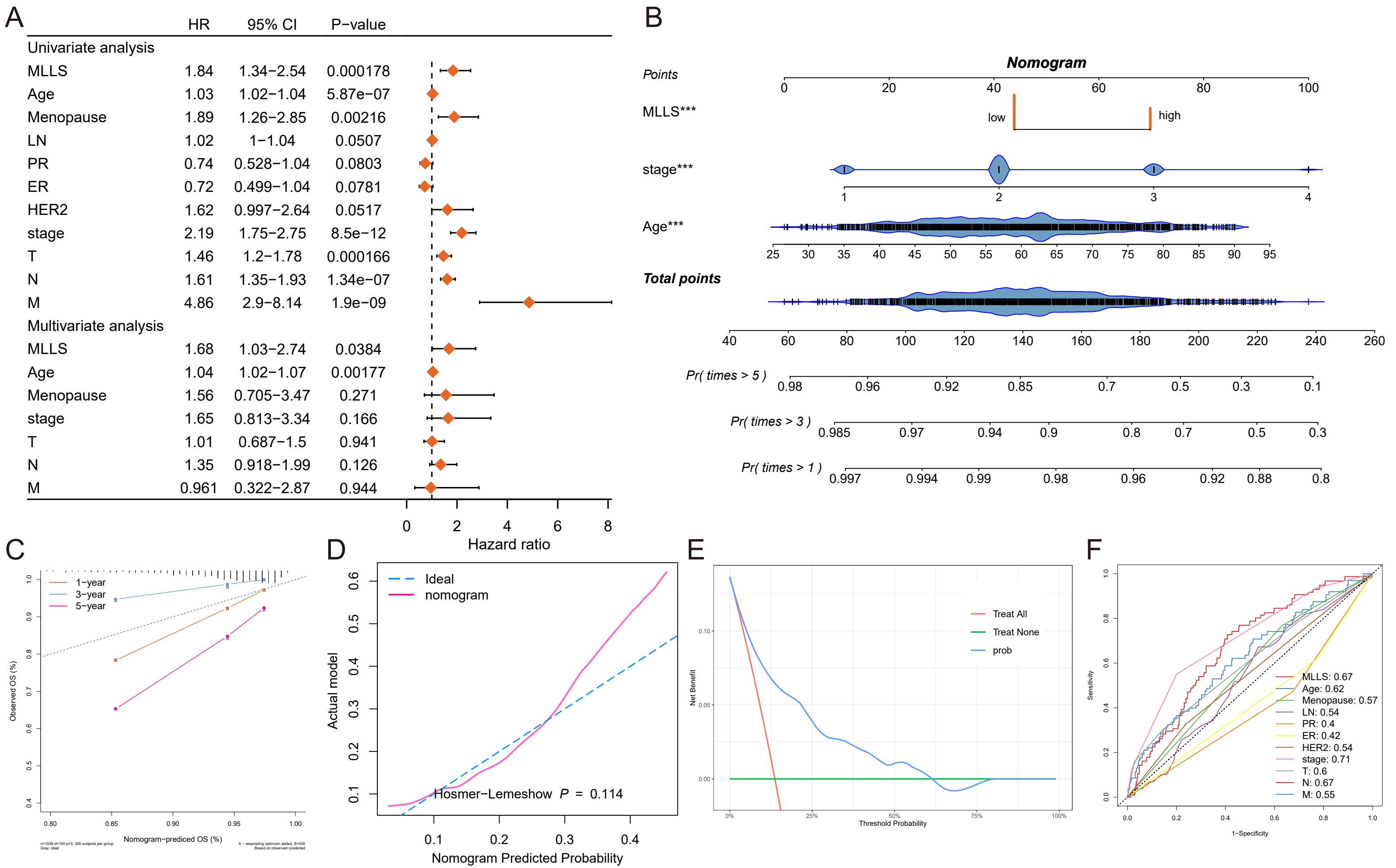

Figure S2. Prognostic characters of MLLS. (A) Forest plot illustrates the results of univariate and multivariate analyses, detailing hazard ratios (HR), 95% confidence intervals (CI), and p-values for various prognostic factors, providing a statistical view on the impact of these factors on patient outcomes. (B) A nomogram for predicting patient survival based on prognostic factors such as MLTS, stage, and age is shown; here, points are assigned to each factor, summed to a total, with corresponding survival probabilities indicated for different time frames (1, 3, and 5 years), offering a personalized survival prediction tool. (C) The calibration curve for the nomogram plots the nomogram-predicted survival probabilities on the x-axis against the observed survival rates at 1, 3, and 5 years on the y-axis, serving as a validation tool for the nomogram's accuracy. (D) Decision curve analysis (DCA) is displayed, showing the net benefit across a range of threshold probabilities for three different strategies: treating all patients, treating none, or using the MLLS score to guide treatment decisions, helping to evaluate the clinical usefulness of the MLLS score in making treatment decisions. (E) Receiver operating characteristic (ROC) curves for various factors used in the nomogram demonstrate the trade-off between sensitivity and specificity for predicting patient outcomes, aiding in assessing the predictive performance of the nomogram. (F) A kernel-smoothing hazard function plot estimates the recurrence rate for different MLLS subgroups, providing insights into the risk dynamics within these subgroups over time.
