# Supplementary figures and images for: Personalized treatment decision-making using a machine learning-derived lactylation signature for breast cancer prognosis
Source: Front Immunol. 2025 May 8;16:1540018. doi: 10.3389/fimmu.2025.1540018 (PMC12095166; doi:10.3389/fimmu.2025.1540018)

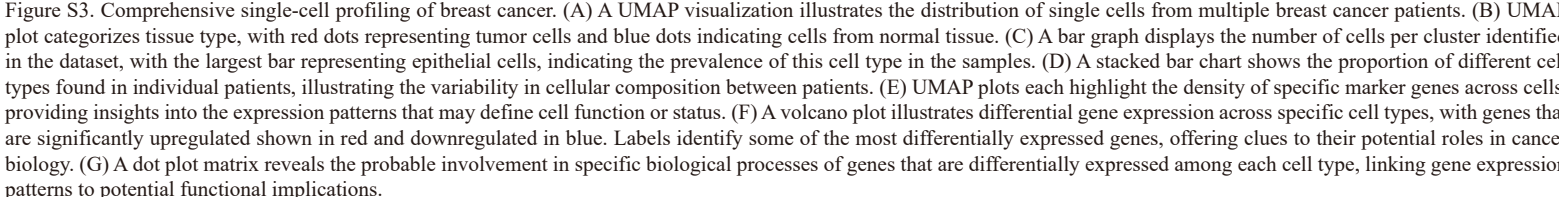

Supplement: Supplementary file 3 [file DataSheet3.pdf]
