# Supplementary material for: Personalized treatment decision-making using a machine learning-derived lactylation signature for breast cancer prognosis
Source: Front Immunol. 2025 May 8;16:1540018. doi: 10.3389/fimmu.2025.1540018 (PMC12095166; doi:10.3389/fimmu.2025.1540018)
Supplement: Supplementary file 4 [file DataSheet4.pdf]

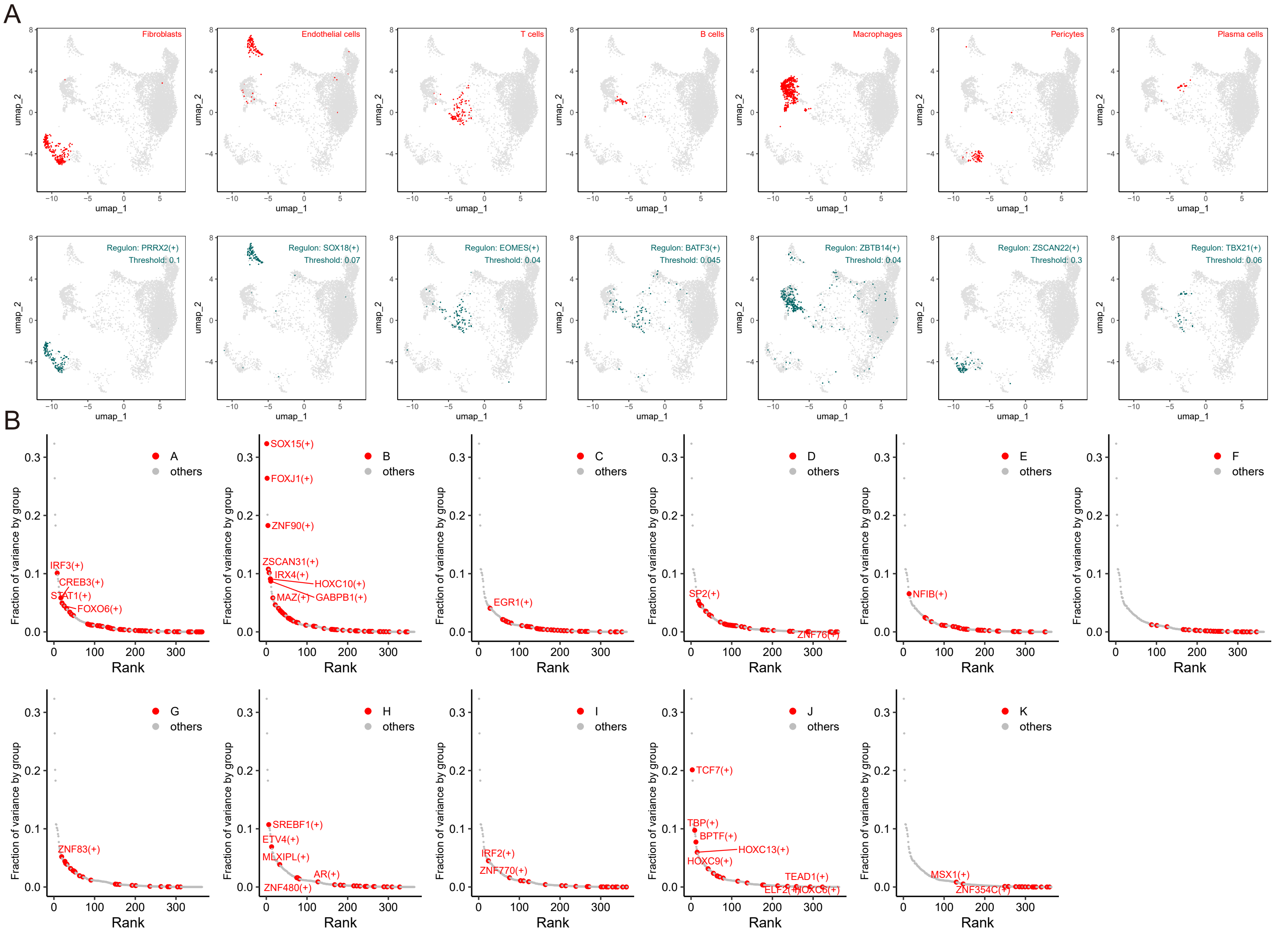

Figure S4. Transcription factor activity correlation and contribution analysis in cell types. (A) UMAP projections categorize samples by transcription factor activity, revealing distinct cell types based on their transcriptional profiles, aiding in the identification of unique cellular behaviors and properties. (B) Illustrates the contribution of different transcription factor groups to MLLS, with significant transcription factors highlighted and ranked based on their RSS, showcasing their regulatory impact on gene expression particularly in epithelial cells.
